# How Narcissistic Leaders Impact on Subordinate’s Followership During the COVID-19? The Moderating Role of Organizational Identification

**DOI:** 10.3389/fpsyg.2022.858779

**Published:** 2022-03-15

**Authors:** Lin Wang, Qun Guo

**Affiliations:** ^1^School of Economics & Management, Foshan University, Foshan, China; ^2^Hubei University of Economics, Wuhan, China

**Keywords:** narcissistic leader, followership, self-interested behavior, organizational identification, leadership

## Abstract

The COVID-19 pandemic gave rise to social and economic problems and pose a threat to most of enterprise. Faced with crisis and challenge, effective leaders and devoted employees are important factors for enterprises to overcome difficulties. We propose a moderated mediation model wherein narcissistic leader predicts subordinate’s followership through leader self-interest behavior perceived by subordinates, with organizational identification of leader acting as the contextual condition. Two-wave data collected from 303 employees in the manufacturing and technology industry in China supported our hypothesized model. We found that narcissistic leader has negative impact on subordinates’ followership due to their perception of leader’s self-interest behavior. Further, organizational identification of leader plays a moderate role in the relationship between narcissistic leader and subordinates’ followership. Theoretical and practical implications are discussed. We also offer several promising directions for future research.

## Introduction

The sudden COVID-19 pandemic caught a large number of companies facing crisis, in the uncertain external environment, subordinates hope to have a effective leader who can lead the enterprise to overcome difficulties and thereby enhance their psychological security (Abbas et al., 2021a). However, for companies and leaders, subordinate with high followership is a significant factor for stabilizing organizational performance (Whitlock, 2013). Scholars have found that positive styles of leadership, such as authentic leadership, transformational leadership, charismatic leadership and servant leadership play a positive effect on the followership of subordinates (Miller et al., 2004; Leroy et al., 2015; Abbas et al., 2020, 2021c). However, compared with a more certain external environment, individuals with narcissistic traits are more likely to become leaders in the case of uncertainty and show more effectiveness in crisis management (Nevicka et al., 2013; Watts et al., 2013). But for narcissistic leaders, whether they are infinitely charismatic or so annoying remains to be seen. Leadership research needs more perspectives, the narcissism of organizational leaders should not be overlooked, even though transformational leaders have the power to affect others in positive ways. When a company’s executives are afflicted with narcissism, it can cause the organization to run at a dangerously high level, accelerating or exacerbating its downfall (Wowak et al., 2017).

Most studies show that narcissism in leaders can be detrimental to employees and their organizations. For instance, narcissistic leaders will encroach on employees’ rights and interests, weaken employees’ autonomy and boost their pressure (Nevicka et al., 2018a), and often make employees intimidated due to negative aspects of arrogance, hostility and manipulation (Leckelt et al., 2015), lead to counter-productive work behavior and workplace deviation of subordinates (Penney and Spector, 2010), keep down honesty in communication and subordinates’ trust in leaders (Benson and Hogan, 2008). However, some scholars have found out that narcissistic leaders can win the followership of their subordinates by their acts of humility (Owens et al., 2015), and motivate their employees with enthusiastic and spiritual description, making the employees sincerely committed to the leader and dedicated to the organization (Rosenthal and Pittinsky, 2006). Thus, it can be seen that there are inconsistent results for the impact of narcissistic leaders on employees’ attitude and behavior, and more empirical studies are needed to verify the impact of narcissistic leaders on subordinates’ behavior (Owens et al., 2015).

Narcissistic leaders are egoists who take use of the leading power strictly in accordance with their own interests, and the important motivation for their behaviors is to satisfy their own needs. In addition to serving the organization, in most cases they use all available resources to improve themselves, and think of it as a way to gain their own sense of superiority (Resick et al., 2009). Studies from some scholars have confirmed that there is a negative relationship between narcissism and altruistic behavior (Peterson et al., 2012). There are also studies indicating that when narcissistic leaders encounter unfair treatment, self-interested behavior will be stimulated (Liu et al., 2017). In conclusion, this study aims to find ways to reduce the negative impact of self-interested behavior on subordinates.

Previous studies have proved that narcissistic leaders have strong self-centered and self-interested motives (Williams, 2014). Thus, even in the absence of external environmental stimulation, they will still take certain self-interested behavior for the sake of self-interest or self-improvement, making it quite necessary to explore what factors can reduce their self-interested motivation or self-interested behavior. Studies have shown that a high organizational identification can lead individuals to produce behavior and desire to assist members of the organization (Blader et al., 2007; Boivie et al., 2011). When a leader has a high organizational identification, he or she will give priority to organizational goals and benefits, this seems to run counter to the trait that narcissistic leaders pursue self-improvement and value self-interest. But in reality, the narcissistic and popular leaders are all in their own success and lead the enterprise to brilliant success in meantime. We have seen there are more identity in leaders and organizations, and such identity is the core of organizational identification (van Knippenberg and Sleebos, 2006). Especially during the COVID-19, the leader’s organizational identity is very important for the development of enterprise. Holmes et al. (2021) referred that when studying the impact of executive personality on an organization, the combined effects of leaders, teams, and environmental factors should be considered.

The outbreak of COVID-19 pandemic has caused certain changes in people’s living habits and thinking habits, including employees’ perception of the work environment (Spagnoli et al., 2020). Scholars have focused on the impact of positive leadership on employee attitudes and behavior during the COVID-19 pandemic (Um-e-Rubbab et al., 2021). However, the impact of leaders with a dark side such as narcissistic leaders on organizations and employees has not been fully discussed, especially in the context of collectivist culture. Thus, from the perspective of social identity theory, this study explores whether narcissistic leaders will lose the followership because of self-interested behavior during the COVID-19, and based on this, discusses the moderating effect of organizational identification in leaders.

## Literature Review and Hypotheses Development

### Narcissistic Leaders and Followership

Scholars considering that when the leader’s behavior is not based on the organizational benefits, but driven by his or her extremely selfish personal ideas and needs, he or she is just narcissistic leaders (Rosenthal and Pittinsky, 2006). Studies show that despite narcissistic leaders attract some followers under certain circumstances, their relationship will be rapidly deteriorating in the subsequent contact after leaving a relatively good first impression, instead it brings about serious discomfort (Rosenthal and Pittinsky, 2006). Kouzes and Posner (1995) believed that honesty, foresight, competence and charisma of leaders are the main reasons for the followership of subordinates. However, even though narcissistic leaders may have strong social ability, extraordinary vision and charming appearance that lead some people to follow them (Rosenthal and Pittinsky, 2006), this followership is only superficial and short-lived, which cannot bring effective improvement of followership. Scholars believe that subordinates’ identification with their leaders can positively predict their loyalty to their leaders (Knippenberg et al., 2004). However, narcissistic leaders just care about themselves but do not care for subordinates, and often resort to fraud to satisfy their own interests, as well as taking improper supervision to maintain their own rights and interests and authority (Nevicka et al., 2018b). In this case, it is difficult for subordinates to have identity with the leader, and improper supervision by the leader will have a series of negative effects on followers. Previous studies have confirmed the negative effects of improper supervision by the leader on followership (Ding and Zhang, 2013). Thus, the following hypothesis is proposed:

*H1: Narcissistic Leaders have negative impact on subordinates’ followership.*

### The Mediating Role of Self-Interested Behavior Perceived by Subordinates

Self-interest occurs as an important motive for human behavior. Decelles et al. (2012) defined self-interested behavior as behavior that is selfish and conducted at the cost of common interests. Camps et al. (2012) believed that the self-interest of leaders is a subjective experience or feeling from subordinates, and whether a leader’s behavior is self-interested or not depends on the subordinates’ subjective cognition and assessment. Self-interested behavior from leaders is a negative behavior, which may own a certain concealment in avoidance of being discovered by subordinates and organizations. Thereby, the concept with regard to self-interested behavior of leaders that Camps et al. (2012) proposed has been adopted in this study: self-interested behavior is the extent to which the subordinates perceive that the leader places his or her material benefits and interests before employee needs and organizational goals.

Despite there is no direct evidence for a connection between narcissism and self-interest, some studies have found a negative relationship between narcissism and altruistic behavior (Schmid et al., 2016). Nevicka et al. (2011) argued that narcissistic leaders are egoists who exercise leading power centering on self-interest. They are skilled in making full use of all available resources to win respect from others, and take this as an important way to gain a sense of superiority. Thus, narcissistic leaders may be believed to try to obtain what they think they are qualified to achieve in a self-interested way. There have been studies also showing that narcissistic leaders often execute their power based on individual purpose or self-interested motivation (Khoo and Burch, 2008). When narcissistic leaders are granted certain power, they may conduct more self-interested behaviors with the power to meet their needs of status and self-esteem (Blickle et al., 2010). Moreover, compared with other leadership styles, it is easier for narcissistic leaders to owe the success of the organization or the team to themselves and make the adverse results attributed to others (Hoffman et al., 2011). Furthermore, the high psychological entitlement led by narcissistic traits makes it easier for narcissistic leaders to provide moral licensing for their self-interested behavior (Yam et al., 2017), which increases the possibility of conducting self-interested behavior to a certain extent.

Followership is a kind of relationship, capability or status arising from the interaction with leaders, which is the dynamic performance of followers (Uhl-Bien, 2011). When the subordinates have perception of the leader’s self-interested behavior, it is hard to have identity with the role of “leader-follower,” and the identity with the leader forms the basis for the leader-follower relationship and the influence on individual behavior (Hogg and Terry, 2000). Studies have shown that the self-interested behavior from leaders decreases organizational commitment and cooperative behavior from subordinates (Decoster et al., 2014b; Schmid et al., 2016), leads to the reduction of staff psychological security and the behavior of knowledge concealment (Peng et al., 2019), and undermines the trust from subordinates, resulting in negative reactions from subordinates, and even vindictive acts against the supervisor (Decoster et al., 2014b). It can be seen from the above findings that leaders’ self-interested behavior is a typical negative behavior, which leads to some negative reactions from subordinates. Nevertheless, when the subordinates perceive the self-interested behavior that narcissistic leaders have, they tend to magnify their negative traits such as ego and arrogance, act against narcissistic leaders, and even attribute their loss and the collective loss to narcissistic leaders. These factors can reduce the subordinates’ intention to follow narcissistic leaders. Thus, it can be seen that the self-interested behavior of narcissistic leaders perceived by subordinates brings about the negative relationship between narcissistic leaders and the followership of subordinates to some extent. Based on the above, hypothesis 2 is proposed:

*H2: Leader self-interest behavior perceived by subordinates mediates the negative relationship between narcissistic leaders and subordinates*’ *followership.*

### The Moderating Role of Organizational Identification

The organizational identification of a leader stands for the “identity” with the organization perceived by the leader (Ashforth and Mael, 1989). According to social identity theory, the more leaders identify with the organization, the more they will accept the values and rules of the organization and act in accordance with them. Though for narcissistic leaders, satisfying their own needs always comes first, self-interest and organizational interest are not always opposite. Some scholars argue that narcissistic leaders and organizational identification can sometimes coexist (Reina et al., 2014). When narcissistic leaders have a high organizational identification, they will balance their own goals and organizational goals, and try their best efforts to reduce the loss or adverse effect of individual behavior on the organization, and even make the organizational benefits highly integrated with their individual goals and interests (Dutton et al., 1994), and satisfy the needs of self-improvement during the promotion of organizational development. In such case, self-interested behavior is identical with organizational behavior, which imperceptibly reduces the self-interested behavior perceived by narcissistic leaders. Besides, narcissistic leaders with high organizational identification usually propose more visions that seem to be socialized, thus they are regarded as people who own group consciousness (Deluga, 1997), thus greatly reducing the risk of discovering their self-interested behavior by subordinates. By contrast, when narcissistic leaders possess low organizational identification, they tend to come up with more visions that seem to be individualized or focused on self-interest during working, making the subordinates clearly feel that the leader behaves only for the sake of their own interests. In addition, when narcissistic leaders possess high organizational identification, they can make self-interest and organizational benefits well integrated, thus making it difficult to distinguish whether their behavior serves themselves or the organization, and making the self-interested behavior of narcissistic leaders show more concealment. Thereby, hypothesis 3 is proposed:

*H3: Organizational identification moderates the relationship between narcissistic leaders and self-interest behavior. Such relationship is more pronounced when organizational identification of narcissistic leader is high rather than low.*

**FIGURE 1:**
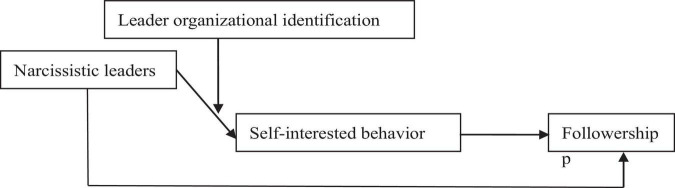
Research framework.

Galvin et al. (2010) believed that narcissistic leaders pursue individualization and personal goals in themselves. The theoretical framework is shown in Figure 1. However, according to Camps et al. (2012), leaders’ self-interested behavior is subjectively perceived by subordinates. When a leader owns self-interested behavior that is not perceived by the subordinates, he or she may not be considered self-interested (Camps et al., 2012). Thus, organizational identification of leaders can, on the one hand, reduce the self-interested behavior from leaders. On the other hand, it can also make self-interested behavior appear to be beneficial to the organization, which makes it difficult for the subordinates to distinguish whether the leader’s behavior is conducted for the sake of themselves or the organization, thus making the self-interested behavior of narcissistic leaders show more concealment. Therefore, the organizational identification of narcissistic leaders can reduce the negative effect brought by narcissistic leaders to a certain extent, and reduce the self-interested behavior perceived by subordinates. When the organizational identification of leaders is low, the subordinates feel more self-interested behaviors from leaders, which brings about more negative effects on the followership of subordinates from the narcissistic leaders. However, when they own high organizational identification, the subordinates find it hard to perceive the self-interested behavior from leaders, thus the negative effect on the followership of subordinates is less likely to be led by self-interested behavior. Thereby, hypothesis 4 is proposed.

Thus we have explained how narcissistic leader effect on subordinates’ followership via leader self-interested behavior perceived by subordinates, and propose the moderating role of organizational identification on the narcissistic leader–self-interested behavior relation. Taking these together, we further propose the moderated mediation model of these relationships. Narcissistic leaders with high-quality organizational identification are less likely to be negative impact on subordinate followership due to leader self-interested behavior perceived by subordinates. However, the negative association between narcissistic leader and subordinate followership via leader self-interested behavior is more salient when leaders have low-quality organizational identification, Accordingly, we put forward:

*H4: Organizational identification moderates the negative relationship between narcissistic leader and subordinate followership via self-interested behavior. Such relationship is more pronounced when leader organizational identification is low rather than high.*

## Methodology

### Sampling and Procedure

Purposive sampling was adopted in the research. We conducted a two-phase questionnaire survey by collecting responses from employees working in the service industry in China. On the cover page of the questionnaire, we explained the voluntary nature of this survey and assured anonymity and confidentiality to the participants. Firstly, supervisors were asked to assess the narcissistic personality and organizational identification of themselves. Subordinates were asked to assess their perceptions of self-interested behavior from their supervisors, using their current immediate supervisors as referees. We received 139 completed questionnaires, representing the response rate of 87.0% in Time-1. One month later (Time-2), we conducted the second-phase survey, following the same procedures as in Time-1. Respondents provided their ratings on followership. Every questionnaire was marked with a unique code which was recorded in a master file such that the responses received from the two phases can be matched. Finally, we received 101 complete and valid questionnaires for leaders matched with 303 for subordinates, representing an overall response rate of 72.9%. Within the sample of supervisors, 73% were male, the average age was 37, and the average working experience was 7 years. As for employees, 62% were males and 38% are females; the average age of employees was 29; and the average working experience was 3 years.

“Harman single factor test” was used to detect common method deviations. The results showed that for the data of narcissism and organizational identification questionnaire filled by leaders, factor analysis obtained a total of 7 common factors with eigenvalues greater than 1, which explained 69.67% of the variation, and the proportion of the first principal component was 21.82%, indicating that the scale data filled by leaders had no serious homologous error. When the questionnaire data for employees about self-interest behavior and followership is not rotated, seven common factors with eigenvalues greater than 1 were obtained by factor analysis, which explained 69.65% of the variation, and the proportion of the first principal component was 32.72%. The largest factor could not explain most of the variation, indicating that there was no serious homologous error in the employee data of this study.

### Measures

All measures used in this survey were adopted from the established scales. Considering that all of our participants were Chinese, we went through appropriate back translation procedures to develop the Chinese version for the measures.

### Narcissistic Leader

We assessed narcissistic leaders by using the NPI-16 scale (Ames et al., 2006). This questionnaire is self-evaluated by the leader. Sample items such as: “I think I am different.”

### Followership

The 21 items followership scale was adopted, developed based on the Chinese context (Zhou et al., 2015). Sample items is “I admire and learn from the leadership’s ability in business, management, etc.”

### Self-Interested Behavior

We used the 9-item scales from Rus (2009) to measure leader self-interested behavior perceived by subordinates. For example: “The bonuses my leader strives for himself is a lot more than for his subordinates,” etc.

### Organizational Identification

The measurement of organizational identification selects the 6-item scale developed by Mael and Tetrick (1992). Example items such as: “When talking about my organization, I often say ‘we.”’

### Control Variables

Except for the narcissism scale, the measurements of the concepts including followership, self-interested behavior, and organizational identification all use Likert’s 5 evaluation scale, 1 means “completely disagree,” and 5 means “completely agree.”

We controlled for the effects of the sex of leaders and subordinates’ age, gender and organizational tenure. Studies have shown that gender stereotypes will affect employees’ expectations of leadership behavior and styles, and the subordinates’ gender will affect their ability to accept different leadership styles (Eagly and Karau, 2002). Foster et al. (2003) found that the narcissistic behavior of employees in an organization decreases with age through empirical research, which may be due to life experience and self-improvement. In addition, working years may affect the organizational identification of leaders, as well as the attitudes and behaviors of subordinates toward leaders. This study will use the above variables as control variables.

## Results

### Analytical Strategy

Because the study involved two levels of variables, we have to test the reliability of score within group (ICC1), reliability of mean group score (ICC2) and Rwg. The results show that self-interested behavior and followership scales can be analyzed across levels with team-level data. The test results of self-interested behavior as follows: (*F* = 6.248, *P* < 0.001), ICC1 = 0.512, ICC2 = 0.840, Rwg = 0.902. The test results of followership are as follows: (*F* = 5.012, *P* < 0.001), ICC1 = 0.445, ICC2 = 0.800, Rwg = 0.980 > 0.7. ICC1 and ICC2 meet the 0.12 standard recommended by James (1982) and 0.47 standard recommended by Schneider et al. (1998). Rwg > 0.7, indicating that the self-interested behavior and followership scales have a good degree of consensus and reliability.

### Confirmatory Factor Analysis

The Cronbach’s alpha coefficient was used to measure the internal consistency reliability of the questionnaire as a whole and each item. The Cronbach’s coefficients of self-interested behavior, followership and organizational identification are as follows: α_(sb)_ = 0.95, α_(f)_ = 0.90, α_(or)_ = 0.90. Respectively, it can be seen that the reliability coefficient of each scale is greater than 0.7, indicating that each dimension has good internal consistency.

In addition, confirmatory factor analysis was performed on the scale using AMOS 20.0, and the results showed that the organizational identification scale at the team level had a good fit (χ^2^/df = 1.11, GFI = 0.97, NFI = 0.98, CFI = 0.951, RMSEA = 0.03 < 0.08), indicating that this scale has good structural validity. The main fitting indicators of the self-interested behavior and followership two-factor model at the individual level are better than the single-factor model (χ^2^/df = 1.61, GFI = 0.88, NFI = 0.88, CFI = 0.95, RMSEA = 0.045 < 0.08), indicating the two variables in the individual level have a two-factor structure and have good construct validity, as shown in Table 1, suggesting that our respondents could distinguish the focal constructs clearly.

**TABLE 1:** Confirmatory factor analysis result.

	Measurement model	*X* ^2^	df	*X*^2^/df	SRMR	GFI	NFI	CFI	RMSEA
Level-2	I	7.78	7	1.11	0.02	0.97	0.98	0.95	0.03
Level-1	Two-factor (S + F)	638.90	398	1.61	0.04	0.88	0.88	0.95	0.04
	One-factor	2588.43	405	6.39	0.14	0.49	0.52	0.56	0.13

*S, self-interested behavior; I, organizational identification; F, followership; N = 303.*

### Descriptive Statistics and Correlation Analysis

The descriptive statistics and related analysis results of narcissistic leaders, followership, self-interested behavior, and organizational identification of leaders are shown in Table 2. Narcissistic leaders and followership are significantly negatively correlated (*r* = −0.55, *P* < 0.01), and narcissistic leaders have a negative relationship with self-interested behavior and organizational identification. Interested behavior has a significant positive impact (*r* = 0.48, *P* < 0.01), and self-interested behavior has a significant negative correlation with subordinate followership (*r* = −0.61, *P* < 0.01).

**TABLE 2:** Means, standard deviations, and correlations of variables.

	1	2	3	4	5	6	7	8	9	10
L-gender	−									
L-age	−0.25*	−								
L-tenure	−0.16*	0.63**	−							
E-gender	0.37**	–0.15	–0.03	−						*
E-age	–0.15	0.11	0.38**	0.11	−					
E-tenure	−0.28**	0.09	0.37**	–0.07	0.76**	−				
N	0.16	–0.36	0.41*	0.04	–0.05	0.14	−			
F	0.09	−0.32**	−0.22**	0.08	–0.02	–0.07	−0.55**	−		
S	–0.12	0.20**	0.22	–0.08	0.05	0.16	0.48**	−0.61**	−	
I	0.00	0.02	–0.12	0.07	–0.02	–0.02	–0.21	0.32**	−0.38**	-
Mean	1.27	34.54	7.62	1.38	27.88	3.04	7.67	3.73	2.23	2.92
SD	0.45	4.94	5.62	0.28	2.73	1.51	3.20	0.37	0.73	0.70

*p < 0.05, **p < 0.01, n = 303.

*N, narcissistic leaders; S, self-interested behavior; I, organizational identification; F, followership; N = 303.*

### Hypothesis Testing

#### The Mediation Effect Test of Self-Interested Behavior

We used HLM for statistical analysis to examine the mechanism of the influence that team-level narcissistic leaders have on individual-level followership. First, a zero model test was performed on self-interested behavior and followership variables. The intra-group correlation coefficient and between-group variation coefficient of self-interested behavior scales were as follows: *U* = 0.45, *R* = 0.25, ICC = 0.64, χ^2^_(df)_ = 624.82, *p* < 0.001. The inter-group variation accounted for 64% of the total variation, indicating significant differences between the groups. Similarly, the inter-group variation of followership accounted for 57% of the total variation, indicating that subsequent cross-layer analysis can be performed.

It can be seen from Table 3 that narcissistic leaders have a significant negative impact on followership (β = −0.06, *p* < 0.001), hypothesis 1 was supported. Narcissistic leaders have significant positive impact on self-interested behavior (β = 0.10, *p* < 0.01), and self-interested behavior has significant negative impact on followership (β = −0.13, *p* < 0.01). As can be seen from Table 3, compared with model F1, model F2 has a reduced regression coefficient of narcissistic leaders to followership (β = −0.05, *p* < 0.001). Therefore, self-interested behavior has a partial mediating effect between narcissistic leaders and followership, hypothesis 2 was supported.

**TABLE 3:** The mediation effect test of self-interested behavior.

		S		F		
		(S0)	S1	(F0)	F1	F2
Intercept		2.23**	2.23**	3.73**	3.73***	3.73**
Level-1	E_gender		0.00		0.02	0.02
	E_age		−0.03		0.00	−0.01
	E_tenure		0.02		−0.01	0.00
	S					−0.13**
Level-2	L_gender		0.06		0.04	0.05
	L_age		0.00		−0.02*	−0.02*
	L_tenure		0.00		0.01	0.01
	N		0.10**		−0.06***	−0.05***
	R (Sigma-squared)	0.25	0.25	0.08	0.08	0.08
	U (Tau)	0.45	0.34	0.11	0.07	0.05
	Δ*R*^2^		0.11		0.04	0.02
	ICC	0.64	0.58	0.57	0.45	0.37
	Chi-square	624.82***	484.47***	501.23***	328.87***	261.55***
	Deviance	632.75	638.63	264.99	267.33	256.74

*p < 0.05, **p < 0.01, ***p < 0.001, n = 303.

*N, narcissistic leaders; S, self-interested behavior; F, followership; L, leaders; E, employees; N = 303.*

#### The Moderating Effect of Organizational Identification

**FIGURE 2:**
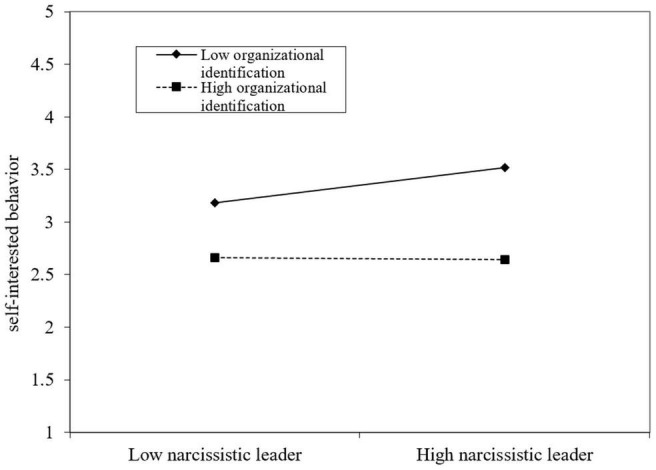
The moderating effect of organizational identification.

After controlling relevant variables such as demographics, our study performed multiple linear regression analysis on the data. The results are shown in Table 4. Model S2 is based on model S1 and included the adjustment variable organizational identification. The results show the percentage of variation between groups decrease from 0.58 to 0.54, organizational identification has a significant negative impact on self-interested behavior (β = −0.32, *p* < 0.01); model S3 is based on model S2 and included N × I interaction terms, the results show that the percentage of variation between groups changed from 0.54 to 0.50, indicating a significant negative impact on self-interested behavior (β = −0.09, *p* < 0.01), further proving that organizational identification has a significant regulatory effect. In order to further prove that the moderating effect of organizational identification has on narcissistic leaders and self-interested behavior is as expected, we draw a diagram of the moderating effect as shown in Figure 2.

**TABLE 4:** The moderating effect of organizational identification.

	S			
	(S0)	S1	S2	S3
Intercept	2.23***	2.23***	2.23***	2.23***
E_gender		0.00	0.01	−0.01
E_age		−0.03*	−0.02*	−0.03
E_tenure		0.02	0.03	0.03
L_gender		−0.06	−0.07	−0.07
L_age		0.00	0.01	0.00
L_tenure		0.00	0.00	0.00
N		0.10***	0.09**	0.08**
I			−0.32**	−0.35**
N × I				−0.09**
R (Sigma-squared)	0.25	0.25	0.25	0.25
U (Tau)	0.45	0.34	0.30	0.25
Δ*R*^2^		0.11	0.05	0.04
ICC	0.64	0.58	0.54	0.50
Chi-square	624.82***	484.47***	427.55***	376.24***
Deviance	632.75	638.63	631.62	623.11

*p < 0.05, **p < 0.01, ***p < 0.001, n = 303.

*N, narcissistic leaders; S, self-interested behavior; I, organizational identification; F, followership; L, leaders; E, employees.*

According to the moderating effect diagram, the influence of narcissistic leader on self-interested behavior is alleviated when the organizational identification is high. However, when organizational identification is low, the influence of narcissistic leaders on self-interest behavior is stronger, Hypothesis 3 was supported.

#### Moderated Mediation Model Test

We used bootstrapping to evaluate the moderated mediation model. The results show that when organizational identification is low (one standard deviation below the mean level), the indirect effect is −0.03, and the Bootstrap result does not contain zero in the 95% confidence interval [−0.06, −0.02], indicating the mediating effect is significant when the moderating variable at low level; when the organizational identification is high (one standard deviation higher than the mean level), the indirect effect is 0.00. Bootstrap result within the 95% confidence interval [−0.02, 0.01] includes zero, which indicates that the mediating effect is not significant when the moderating variable is at a high level, as shown in Table 5. In summary, organizational identification has a significant regulatory effect on this set of mediating effects. The analysis results show that organizational identification weakens the indirect effect of narcissistic leaders on the followership through self-interested behavior: with lower organizational identification of the leader, comes a more significant mediating effect of self-interested behavior on narcissistic leaders and followership, therefore hypothesis 4 was supported.

**TABLE 5:** Moderated mediation model test.

	Organizational identification		Effect coefficient	SD	LLCI	ULCI
Indirect effect	Low	2.22	−0.03	0.01	−0.06	−0.02
	Medium	2.92	−0.02	0.01	−0.03	−0.01
	High	3.63	0.00	0.01	−0.02	0.01

## Discussion and Conclusion

Scholars have found that narcissism plays a positive role in predicting self-interested behavior (Liu et al., 2017), but can the employees perceive the self-interested behavior of the narcissistic leaders and thus change their attitude and behavior toward the leaders? Few studies have investigated the conduction role of self-interested behavior that is perceived by subordinates between leadership traits and subordinates’ attitudes and behaviors. In regard to the moderating factors of leaders’ self-interested behavior, scholars have, respectively, discussed from the aspects of subordinates and external surroundings, and found that the organizational citizenship behavior of subordinates can keep down the leaders’ hindrance stressors, thus reducing the possibility of self-interested behavior from leaders. The external unfair stimulus can increase the self-interested behavior from narcissistic leaders (Camps et al., 2012; Decoster et al., 2014a), but how other factors of leaders themselves affect subordinates’ perception of self-interested behavior from leaders is seldom involved. The findings of this study show that even if there is no external unfair stimulus, narcissistic leaders still have some self-interested motivation and behavior, and the subordinates’ perception of this kind of self-interested behavior, to some extent, leads to the negative effect of narcissistic leaders on followership. Organizational identification from leaders can alleviate the self-interested behavior conducted by narcissistic leaders and regulate the mediating role of self-interested behavior between narcissistic leaders and subordinates’ followership. The organizational identification seems to be contradictory to self-interested behavior, but it is not impossible for them to coexist. Especially during the COVID-19 pandemic, organization and employees are facing many challenges, narcissistic leaders with high organizational identification will fully consider the interests of the organization and bring employees a certain sense of psychological security. The research findings show that when the organizational identification of narcissistic leaders is high, the self-interested behavior can be restrained or concealed, thus mitigating the negative effect on the attitudes and behaviors of subordinates brought by narcissistic leaders.

### Theoretical Implications

Scholars have focused on the impact of leaders with different traits on employee attitudes and behaviors in the context of COVID-19, especially some positive leadership, but lack of arguments that how narcissistic leadership, a two-faced leader, affects employees and organizations during the special period (Um-e-Rubbab et al., 2021). This study reveals the influencing mechanism that narcissistic leaders bring to the followership of subordinates. “Leaders can really get somewhere only with the followers’ identity.” Narcissistic leaders are satisfied with getting vanity from their subordinates’ praise, and need to constantly find themselves from others’ reaction to them, compared with other leaders, they rely more on their subordinates’ following behavior. Most existing studies have explored the influence of positive leadership style on followership on the basis on social exchange theory or self-determination theory (Leroy et al., 2015). How the leaders with dark qualities like narcissistic leaders affect the followership of subordinates is rarely discussed. From the perspective of social identity theory, this study explores the influencing mechanism that complex leadership traits such as narcissistic leaders bring to the followership of subordinates.

From the perspective of subordinates’ perception of leader behavior, it explores the mediating effect of leadership traits on subordinates. At present, most studies on narcissistic leaders view narcissistic leaders as a personality trait. However, as a personality trait, narcissism is a distal cause which influence subordinates’ attitudes and behaviors, which is often conveyed by the proximal cause of leader behavior therein. Currently few studies have explored the mediating effect from this perspective. In this study, the self-interested behavior from leaders perceived by subordinates is regarded as an intermediate variable between narcissistic leaders and followership of subordinates, and the relevant research findings are enriched. Besides, different cultures also have different effects on leader behavior and employee responses (Abbas et al., 2021b). Chinese society has always valued the virtuous role of leaders and emphasized the collectivism culture, while self-interested behavior is a typical behavior that runs contrary to traditional morality and goes against people’s definition of a “good leader” which makes leaders’ self-interested behavior more covert. As a consequence, it seems more necessary to pay attention to the intermediary role of self-interested behavior between leaders and followership in the Chinese context.

It has demonstrated the role of organizational identification and self-interested behavior, which seem to be two contradictory concepts, in the effect of narcissistic leaders. Narcissistic leaders, as a kind of leader with dual personality traits, whose effects on the organization and subordinates are inconsistent in the research findings. This study shows that the behavior from narcissistic leaders and their impact on subordinates depend, to some extent, on how leaders treat their organization. Previous studies paid more attention to the influence of subordinates’ characteristics and external surroundings on narcissistic leaders, and often ignored the joint effect of other factors in themselves. According to the theory of contradiction, though the two aspects in a contradiction seem inconsistent or even negatively correlated, it is necessary to make the two aspects in the contradiction harmonious or unified in order to achieve the effectiveness of management (Smith and Lewis, 2011). Hence, the seemingly opposite behaviors of self-interest and organizational identification are not impossible to coexist. This study has proved the inhibiting effect that the organizational identification of narcissistic leaders themselves brings to the negative effects.

### Practical Implications

Compared with a more certain external environment, narcissistic individuals are more likely to become leaders in uncertain situations (Nevicka et al., 2013), and show stronger skills in crisis management (Watts et al., 2013). But the findings of this study also show that it is easier for narcissistic leaders to have self-interested behavior. Leaders executing power for the sake of self-interest will inevitably bring losses to the enterprise. Thus, on the one hand organizations should give full play to the advantages of narcissistic leaders who dare to take risks and innovate especially in the face of the crisis brought by the COVID-19, and meanwhile it should pay full attention to the negative effect that narcissistic leaders may bring about, do restriction by completed system and rules, and make narcissism imprisoned, so as to prevent narcissistic leaders from harming the collective interests for the sake of personal honor.

Enterprises should place emphasis on improving organizational identification of narcissistic leaders and make them all in the same boat with the organization. The audacity and charisma of narcissistic leaders make it hard to prevent them from being a leader. Thus, in addition to establishing a completed system, it is more urgent to restrain and prevent the negative harm brought by narcissistic leaders. Sometimes the harm brought by narcissistic leaders is intangible or hidden, and some rigid rules can hardly take full control of the “demons” in their heart, thus some soft measures are in need to make them self-restraint. Organizations can improve the organizational identification of narcissistic leaders by constructing organizational culture and organizational atmosphere, and unify their personal interests with organizational interests as far as possible, so as to avoid behaviors detrimental to organizational interests conducted by narcissistic leaders to some extent.

The new crown epidemic has had a great impact on the psychology and behavior of employees. Organizations needs to further focus on the impact of employee followership on organizational development during the COVID-19. Followers decide the success or failure of the enterprise (Kellerman, 1984). The improvement of followership plays a significantly positive effect on subordinates’ work performance. Thus, enterprise leaders should pay full attention to the influence of their own behavior on the followership of subordinates. Particularly the narcissistic leaders should try their best to balance their self-interest and organizational interest, and unify self-set goal with organizational goal as far as possible, so as to reduce the negative effect arising from narcissism, make subordinates feel more about their self-confidence and charisma with a grand vision, and make them follow wholeheartedly instead of superficial flattery only.

### Research Limitations

We conducted a questionnaire survey in two time periods, but it cannot guarantee a good evaluation of the causal relationship between variables. Future research can make a more rigorous test on the research problems through longitudinal data with a large time span or through the introduction of experimental research and other methods, so as to make the relationship between related variables more convincing.

The measurement of narcissistic leaders is understood as a unified one-dimensional construct. Some scholars have noticed that narcissistic leaders contain multiple dimensions, such as knowledge inhibition, charisma or positive narcissism and negative narcissism. However, most of the research is theoretical level, and there can be more empirical research in the future.

For leaders with narcissism, they may have different effects under some special circumstances or in the face of different subordinates and teams with different characteristics. Future research can explore the combined effect of these factors, so as to verify the positive role of narcissistic leaders.

## Data Availability Statement

The raw data supporting the conclusions of this article will be made available by the authors, without undue reservation.

## Ethics Statement

The studies involving human participants were reviewed and approved by the Academic Committee of School of Economics and Management of Foshan University. The patients/participants provided their written informed consent to participate in this study.

## Author Contributions

Both authors listed have made a substantial, direct, and intellectual contribution to the work, and approved it for publication.

## Conflict of Interest

The authors declare that the research was conducted in the absence of any commercial or financial relationships that could be construed as a potential conflict of interest.

## Publisher’s Note

All claims expressed in this article are solely those of the authors and do not necessarily represent those of their affiliated organizations, or those of the publisher, the editors and the reviewers. Any product that may be evaluated in this article, or claim that may be made by its manufacturer, is not guaranteed or endorsed by the publisher.
